# *cis*-2-(4-Meth­oxy­phen­yl)-4-methyl-1,2-di­hydro­naphthalen-1-ol

**DOI:** 10.1107/S1600536814007739

**Published:** 2014-04-12

**Authors:** Alan J. Lough, Mohammed-Abdul Raheem, William Tam

**Affiliations:** a Department of Chemistry, University of Toronto, Toronto, Ontario, M5S 3H6, Canada; b Department of Chemistry, University of Guelph, Guelph, Ontario, N1G 2W1, Canada

## Abstract

The stereochemistry and regiochemistry of the title compound, C_18_H_18_O_2_, were determined by the X-ray analysis. There are two independent mol­ecules in the asymmetric unit in which the dihedral angles between the benzene rings are 88.31 (4) and 86.27 (4)°. The cyclo­hexene rings are in half-chair conformations. In the crystal, O—H⋯O hydrogen bonds link alternating types of mol­ecules into chains along [010] with graph-set *C*_2_^2^(4).

## Related literature   

For metal-catalysed ring-opening reactions of oxanorbornadiene compounds, see: Jack *et al.* (2013 ▶). For hydrogen-bond graph-set notation, see: Bernstein *et al.* (1995 ▶).
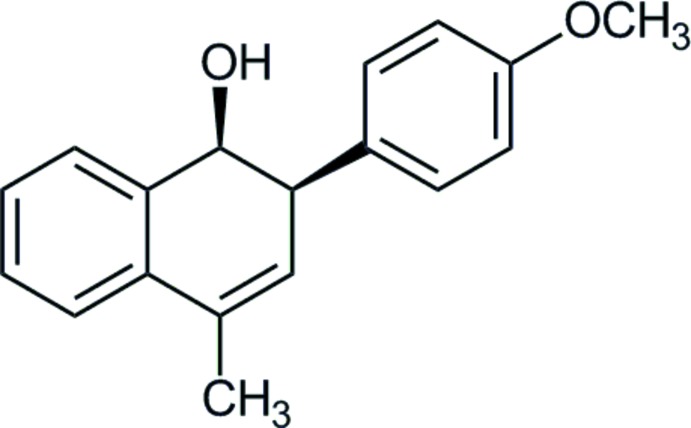


## Experimental   

### 

#### Crystal data  

C_18_H_18_O_2_
*M* *_r_* = 266.32
Orthorhombic, 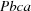

*a* = 11.4550 (3) Å
*b* = 11.2239 (3) Å
*c* = 44.3776 (10) Å
*V* = 5705.6 (2) Å^3^
*Z* = 16
Cu *K*α radiation
μ = 0.63 mm^−1^
*T* = 150 K
0.20 × 0.20 × 0.19 mm

#### Data collection  

Bruker Kappa APEX DUO CCD diffractometer
Absorption correction: multi-scan (*SADABS*; Bruker, 2012 ▶) *T*_min_ = 0.707, *T*_max_ = 0.753
34754 measured reflections
4946 independent reflections
4644 reflections with *I* > 2σ(*I*)
*R*_int_ = 0.033

#### Refinement  

*R*[*F* ^2^ > 2σ(*F* ^2^)] = 0.039
*wR*(*F* ^2^) = 0.099
*S* = 1.05
4946 reflections
373 parameters
H atoms treated by a mixture of independent and constrained refinement
Δρ_max_ = 0.23 e Å^−3^
Δρ_min_ = −0.25 e Å^−3^

### 

Data collection: *APEX2* (Bruker, 2012 ▶); cell refinement: *SAINT* (Bruker, 2012 ▶); data reduction: *SAINT*; program(s) used to solve structure: *SHELXS97* (Sheldrick, 2008 ▶); program(s) used to refine structure: *SHELXL97* (Sheldrick, 2008 ▶); molecular graphics: *PLATON* (Spek, 2009 ▶); software used to prepare material for publication: *SHELXTL* (Sheldrick, 2008 ▶).

## Supplementary Material

Crystal structure: contains datablock(s) I. DOI: 10.1107/S1600536814007739/rn2124sup1.cif

Structure factors: contains datablock(s) I. DOI: 10.1107/S1600536814007739/rn2124Isup2.hkl

Supporting information file. DOI: 10.1107/S1600536814007739/rn2124Isup3.cml

CCDC reference: 995951

Additional supporting information:  crystallographic information; 3D view; checkCIF report

## Figures and Tables

**Table 1:** Hydrogen-bond geometry (Å, °)

*D*—H⋯*A*	*D*—H	H⋯*A*	*D*⋯*A*	*D*—H⋯*A*
O1*A*—H1*O*⋯O1*B*	0.89 (2)	2.23 (2)	3.0346 (15)	150.6 (19)
O1*B*—H2*O*⋯O1*A* ^i^	0.88 (2)	2.04 (2)	2.8973 (15)	163 (2)

Symmetry code: (i) 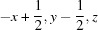.

**Fig. 1.**
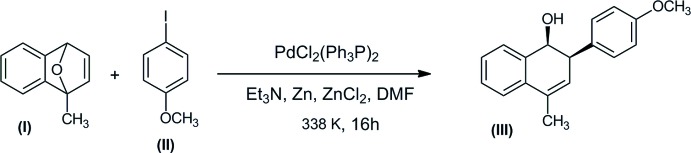
The reaction scheme.

**Fig. 2.**
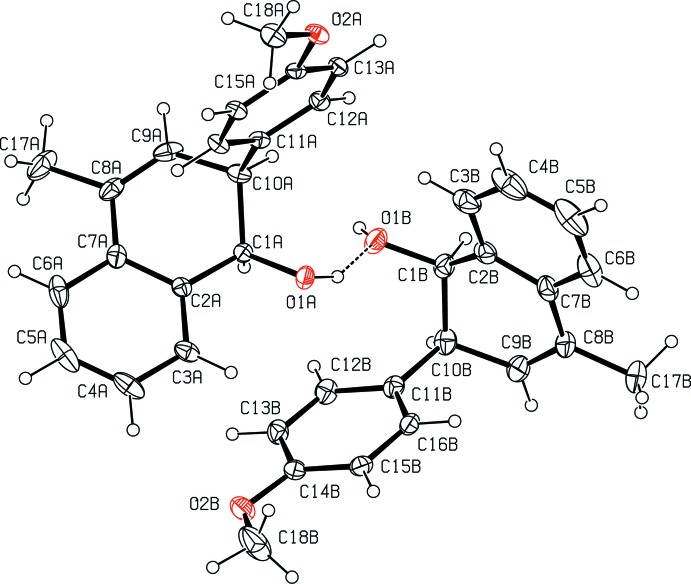
The asymmetric unit of the title compound showing 30% probability ellipsoids. The dashed line indicates a hydrogen bond.

**Fig. 3.**
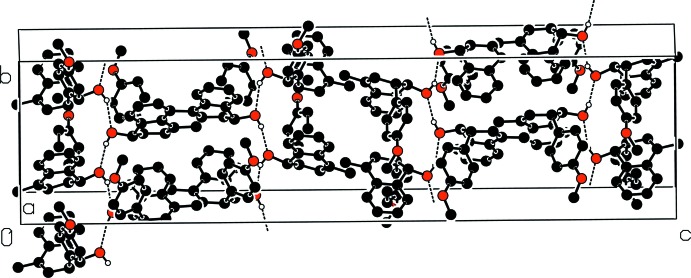
Part of the crystal structure with hydrogen bonds shown as dashed lines.

## supplementary crystallographic information

### 1. Comment

We have recently investigated the metal-catalyzed ring-opening reactions of oxanorbornadiene compounds (Jack *et al.*, 2013). When expanding this reaction using a palladium catalyst, C1-methyl substituted oxanorbornadiene (I) (see Fig. 1) reacts with 4-methoxy-1-iodobenzene (II) in the presence of PdCl_2_(PPh_3_)_2_, Zn, ZnCl_2_, Et_3_N in DMF, to give the ring-opening product (III) as a single regio- and stereoisomer. The stereochemistry and regiochemistry of the product was determined by this single-crystal X-ray analysis. Although different regio- and stereoisomers could be formed, the only ring-opening product obtained was found to have a *cis* stereochemistry.

There are two independent molecules [A and B] in the asymmetric unit which are shown in Fig. 2. The dihedral angles between the two benzene rings [C2–C7/C11–C16] are 88.31 (4) and 86.27 (4)°, for moleclues A and B respetively. The cyclohexene rings [C1/C2/C7—C10] are in half-chair conformations. In the crystal, O—H···O hydrogen bonds link alternating types of molecules into chains along [010] with graph-set C^2^_2_(4) (Bernstein *et al.*, 1995) (see Fig. 3).

### 2. Experimental

C1 methyl substituted oxanorbornadiene (I) (0.50 mmol) and 4-methoxy-1-iodobenzene (II) (0.55 mmol) were added into an oven-dried vial containing PdCl_2_(PPh_3_)_2_ (0.05 mmol), Zn powder (5 mmol), ZnCl_2_ (0.025 mmol), Et_3_N (4 mmol) in DMF (3 ml). The vial was sealed with a screw cap and was heated at 338 K with stirring for 16 h. The crude product was purified by column chromatography (EtOAc: hexanes = 1:4) followed by recrystallization in EtOAc:hexanes = 1:1 to give the *cis* ring-opening (III) in 85% yield. X-ray quality crystals were grown from a solution of the title compound in EtOAc:hexanes = 1:1.

### 3. Refinement

Hydrogen atoms bonded to C atoms were placed in calculated positions with C—H distances ranging from 0.95–1.00 Å and included in the refinement in a riding-model approximation with *U*_iso_(H) = 1.2*U*_eq_(C) or 1.5*U*_eq_(C_methyl_). H atoms bonded to O atoms were refined independently with isotropic displacement parameters.

### Crystal data

**Table:**

C_18_H_18_O_2_	*F*(000) = 2272
*M**_r_* = 266.32	*D*_x_ = 1.240 Mg m^−^^3^
Orthorhombic, *P**b**c**a*	Cu *K*α radiation, λ = 1.54178 Å
Hall symbol: -P 2ac 2ab	Cell parameters from 9819 reflections
*a* = 11.4550 (3) Å	θ = 4.3–66.5°
*b* = 11.2239 (3) Å	µ = 0.63 mm^−^^1^
*c* = 44.3776 (10) Å	*T* = 150 K
*V* = 5705.6 (2) Å^3^	Block, colourless
*Z* = 16	0.20 × 0.20 × 0.19 mm

### Data collection

**Table:**

Bruker Kappa APEX DUO CCD diffractometer	4946 independent reflections
Radiation source: Bruker IµuS	4644 reflections with *I* > 2σ(*I*)
Multi-layer optics monochromator	*R*_int_ = 0.033
φ and ω scans	θ_max_ = 66.5°, θ_min_ = 4.0°
Absorption correction: multi-scan (*SADABS*; Bruker, 2012)	*h* = −13→13
*T*_min_ = 0.707, *T*_max_ = 0.753	*k* = −13→13
34754 measured reflections	*l* = −34→52

### Refinement

**Table:**

Refinement on *F*^2^	Primary atom site location: structure-invariant direct methods
Least-squares matrix: full	Secondary atom site location: difference Fourier map
*R*[*F*^2^ > 2σ(*F*^2^)] = 0.039	Hydrogen site location: inferred from neighbouring sites
*wR*(*F*^2^) = 0.099	H atoms treated by a mixture of independent and constrained refinement
*S* = 1.05	*w* = 1/[σ^2^(*F*_o_^2^) + (0.047*P*)^2^ + 2.0379*P*] where *P* = (*F*_o_^2^ + 2*F*_c_^2^)/3
4946 reflections	(Δ/σ)_max_ = 0.001
373 parameters	Δρ_max_ = 0.23 e Å^−^^3^
0 restraints	Δρ_min_ = −0.25 e Å^−^^3^

### Special details

**Table:**

Geometry. All e.s.d.'s (except the e.s.d. in the dihedral angle between two l.s. planes) are estimated using the full covariance matrix. The cell e.s.d.'s are taken into account individually in the estimation of e.s.d.'s in distances, angles and torsion angles; correlations between e.s.d.'s in cell parameters are only used when they are defined by crystal symmetry. An approximate (isotropic) treatment of cell e.s.d.'s is used for estimating e.s.d.'s involving l.s. planes.
Refinement. Refinement of *F*^2^ against ALL reflections. The weighted *R*-factor *wR* and goodness of fit *S* are based on *F*^2^, conventional *R*-factors *R* are based on *F*, with *F* set to zero for negative *F*^2^. The threshold expression of *F*^2^ > σ(*F*^2^) is used only for calculating *R*-factors(gt) *etc*. and is not relevant to the choice of reflections for refinement. *R*-factors based on *F*^2^ are statistically about twice as large as those based on *F*, and *R*- factors based on ALL data will be even larger.

### Fractional atomic coordinates and isotropic or equivalent isotropic displacement parameters (Å^2^)

**Table:**

	*x*	*y*	*z*	*U*_iso_*/*U*_eq_	
O1A	0.22919 (8)	0.26848 (9)	0.120623 (19)	0.0325 (2)	
O2A	0.68544 (7)	0.53156 (8)	0.07521 (2)	0.0302 (2)	
C1A	0.20394 (10)	0.22033 (10)	0.09162 (3)	0.0238 (2)	
H1AA	0.1625	0.1430	0.0949	0.029*	
C2A	0.12087 (10)	0.30080 (10)	0.07497 (3)	0.0236 (2)	
C3A	0.04550 (10)	0.37600 (11)	0.09018 (3)	0.0319 (3)	
H3AA	0.0512	0.3831	0.1115	0.038*	
C4A	−0.03824 (12)	0.44124 (12)	0.07476 (4)	0.0453 (4)	
H4AA	−0.0899	0.4922	0.0855	0.054*	
C5A	−0.04615 (13)	0.43192 (14)	0.04395 (4)	0.0525 (4)	
H5AA	−0.1024	0.4777	0.0333	0.063*	
C6A	0.02753 (13)	0.35617 (14)	0.02840 (3)	0.0458 (4)	
H6AA	0.0207	0.3499	0.0071	0.055*	
C7A	0.11210 (11)	0.28850 (12)	0.04346 (3)	0.0301 (3)	
C8A	0.18971 (12)	0.20407 (13)	0.02778 (3)	0.0375 (3)	
C9A	0.28302 (12)	0.16040 (12)	0.04187 (3)	0.0366 (3)	
H9AA	0.3316	0.1063	0.0312	0.044*	
C10A	0.31604 (10)	0.19198 (11)	0.07382 (3)	0.0279 (3)	
H10A	0.3507	0.1188	0.0830	0.034*	
C11A	0.40984 (10)	0.28772 (10)	0.07467 (3)	0.0248 (3)	
C12A	0.51443 (10)	0.26726 (11)	0.08995 (3)	0.0270 (3)	
H12A	0.5242	0.1945	0.1006	0.032*	
C13A	0.60418 (10)	0.34949 (11)	0.09013 (3)	0.0268 (3)	
H13A	0.6744	0.3331	0.1008	0.032*	
C14A	0.59110 (10)	0.45625 (10)	0.07467 (3)	0.0237 (2)	
C15A	0.48724 (10)	0.48025 (11)	0.05977 (3)	0.0256 (3)	
H15A	0.4769	0.5540	0.0496	0.031*	
C16A	0.39840 (10)	0.39589 (11)	0.05975 (3)	0.0271 (3)	
H16A	0.3280	0.4127	0.0493	0.033*	
C17A	0.15995 (16)	0.1685 (2)	−0.00402 (3)	0.0680 (6)	
H17A	0.2182	0.1117	−0.0114	0.102*	
H17B	0.1597	0.2394	−0.0169	0.102*	
H17C	0.0826	0.1313	−0.0044	0.102*	
C18A	0.67564 (12)	0.63997 (12)	0.05877 (3)	0.0373 (3)	
H18A	0.7487	0.6848	0.0604	0.056*	
H18B	0.6114	0.6875	0.0670	0.056*	
H18C	0.6600	0.6222	0.0375	0.056*	
O1B	0.27739 (10)	0.01439 (10)	0.14040 (2)	0.0434 (3)	
O2B	−0.16899 (8)	0.31196 (9)	0.14391 (2)	0.0425 (2)	
C1B	0.28972 (12)	−0.00485 (12)	0.17196 (3)	0.0346 (3)	
H1BA	0.3294	−0.0835	0.1746	0.042*	
C2B	0.36914 (12)	0.08848 (11)	0.18513 (3)	0.0341 (3)	
C3B	0.44909 (13)	0.14936 (14)	0.16753 (4)	0.0484 (4)	
H3BA	0.4478	0.1393	0.1463	0.058*	
C4B	0.53099 (15)	0.22476 (16)	0.18035 (6)	0.0670 (6)	
H4BA	0.5853	0.2659	0.1680	0.080*	
C5B	0.53336 (16)	0.23978 (17)	0.21103 (6)	0.0697 (6)	
H5BA	0.5902	0.2903	0.2200	0.084*	
C6B	0.45262 (16)	0.18112 (15)	0.22903 (4)	0.0569 (5)	
H6BA	0.4542	0.1929	0.2502	0.068*	
C7B	0.36911 (12)	0.10518 (12)	0.21653 (3)	0.0382 (3)	
C8B	0.28066 (13)	0.04384 (15)	0.23491 (3)	0.0445 (4)	
C9B	0.19059 (14)	−0.00945 (15)	0.22167 (3)	0.0457 (4)	
H9BA	0.1347	−0.0477	0.2342	0.055*	
C10B	0.17150 (12)	−0.01309 (12)	0.18803 (3)	0.0362 (3)	
H10B	0.1385	−0.0934	0.1832	0.043*	
C11B	0.08270 (11)	0.07827 (12)	0.17737 (3)	0.0306 (3)	
C12B	0.01297 (12)	0.05211 (12)	0.15228 (3)	0.0347 (3)	
H12B	0.0224	−0.0221	0.1423	0.042*	
C13B	−0.06856 (12)	0.13136 (12)	0.14185 (3)	0.0344 (3)	
H13B	−0.1144	0.1118	0.1247	0.041*	
C14B	−0.08461 (10)	0.24027 (12)	0.15619 (3)	0.0317 (3)	
C15B	−0.01645 (11)	0.26885 (12)	0.18096 (3)	0.0325 (3)	
H15B	−0.0264	0.3431	0.1909	0.039*	
C16B	0.06686 (11)	0.18773 (12)	0.19112 (3)	0.0314 (3)	
H16B	0.1141	0.2082	0.2079	0.038*	
C17B	0.29053 (18)	0.0501 (2)	0.26884 (4)	0.0735 (6)	
H17D	0.2271	0.0040	0.2780	0.110*	
H17E	0.2851	0.1333	0.2754	0.110*	
H17F	0.3658	0.0169	0.2752	0.110*	
C18B	−0.18281 (18)	0.42644 (19)	0.15585 (5)	0.0792 (7)	
H18D	−0.2433	0.4690	0.1445	0.119*	
H18E	−0.1089	0.4699	0.1543	0.119*	
H18F	−0.2059	0.4207	0.1771	0.119*	
H1O	0.241 (2)	0.207 (2)	0.1329 (5)	0.077 (7)*	
H2O	0.266 (2)	−0.055 (2)	0.1314 (5)	0.079 (7)*	

### Atomic displacement parameters (Å^2^)

**Table:**

	*U*^11^	*U*^22^	*U*^33^	*U*^12^	*U*^13^	*U*^23^
O1A	0.0338 (5)	0.0414 (5)	0.0223 (4)	−0.0019 (4)	−0.0033 (4)	0.0013 (4)
O2A	0.0198 (4)	0.0288 (5)	0.0419 (5)	−0.0024 (3)	−0.0011 (3)	−0.0038 (4)
C1A	0.0234 (6)	0.0240 (6)	0.0240 (6)	−0.0021 (5)	0.0002 (4)	−0.0002 (5)
C2A	0.0202 (5)	0.0229 (6)	0.0277 (6)	−0.0057 (5)	−0.0013 (4)	0.0005 (5)
C3A	0.0221 (6)	0.0278 (6)	0.0458 (7)	−0.0033 (5)	−0.0023 (5)	−0.0082 (6)
C4A	0.0243 (7)	0.0265 (7)	0.0851 (12)	−0.0006 (5)	−0.0112 (7)	−0.0047 (7)
C5A	0.0326 (7)	0.0362 (8)	0.0887 (13)	−0.0068 (6)	−0.0261 (8)	0.0224 (8)
C6A	0.0402 (8)	0.0541 (9)	0.0432 (8)	−0.0220 (7)	−0.0191 (6)	0.0214 (7)
C7A	0.0275 (6)	0.0350 (7)	0.0278 (6)	−0.0148 (5)	−0.0039 (5)	0.0052 (5)
C8A	0.0348 (7)	0.0507 (8)	0.0270 (6)	−0.0223 (6)	0.0088 (5)	−0.0091 (6)
C9A	0.0335 (7)	0.0334 (7)	0.0430 (7)	−0.0125 (6)	0.0161 (6)	−0.0170 (6)
C10A	0.0240 (6)	0.0208 (6)	0.0390 (7)	0.0008 (5)	0.0038 (5)	−0.0009 (5)
C11A	0.0215 (6)	0.0239 (6)	0.0289 (6)	0.0020 (5)	0.0038 (4)	−0.0036 (5)
C12A	0.0275 (6)	0.0249 (6)	0.0287 (6)	0.0051 (5)	0.0012 (5)	0.0008 (5)
C13A	0.0220 (6)	0.0303 (6)	0.0280 (6)	0.0052 (5)	−0.0031 (5)	−0.0042 (5)
C14A	0.0184 (5)	0.0260 (6)	0.0268 (6)	0.0004 (4)	0.0031 (4)	−0.0075 (5)
C15A	0.0221 (6)	0.0227 (6)	0.0321 (6)	0.0026 (5)	0.0002 (5)	−0.0001 (5)
C16A	0.0190 (5)	0.0274 (6)	0.0350 (6)	0.0023 (5)	−0.0026 (5)	−0.0006 (5)
C17A	0.0593 (10)	0.1120 (16)	0.0326 (8)	−0.0406 (11)	0.0099 (7)	−0.0248 (9)
C18A	0.0305 (7)	0.0323 (7)	0.0492 (8)	−0.0085 (5)	−0.0003 (6)	0.0011 (6)
O1B	0.0589 (7)	0.0453 (6)	0.0261 (5)	0.0162 (5)	0.0019 (4)	−0.0020 (4)
O2B	0.0304 (5)	0.0483 (6)	0.0487 (6)	0.0054 (4)	−0.0065 (4)	−0.0057 (5)
C1B	0.0446 (8)	0.0313 (7)	0.0281 (6)	0.0090 (6)	−0.0011 (6)	0.0035 (5)
C2B	0.0326 (7)	0.0308 (7)	0.0389 (7)	0.0092 (5)	−0.0014 (5)	0.0077 (6)
C3B	0.0366 (8)	0.0499 (9)	0.0588 (9)	0.0091 (7)	0.0044 (7)	0.0215 (7)
C4B	0.0375 (9)	0.0526 (10)	0.1108 (17)	−0.0023 (8)	−0.0066 (10)	0.0348 (11)
C5B	0.0470 (10)	0.0444 (10)	0.1175 (18)	−0.0057 (8)	−0.0304 (11)	0.0114 (11)
C6B	0.0571 (10)	0.0474 (9)	0.0662 (11)	0.0103 (8)	−0.0288 (9)	−0.0056 (8)
C7B	0.0391 (7)	0.0367 (7)	0.0387 (7)	0.0095 (6)	−0.0099 (6)	0.0029 (6)
C8B	0.0475 (9)	0.0569 (9)	0.0290 (7)	0.0110 (7)	−0.0045 (6)	0.0084 (6)
C9B	0.0472 (8)	0.0562 (9)	0.0338 (7)	0.0015 (7)	0.0033 (6)	0.0201 (7)
C10B	0.0424 (8)	0.0319 (7)	0.0344 (7)	−0.0059 (6)	−0.0031 (6)	0.0070 (5)
C11B	0.0312 (6)	0.0344 (7)	0.0262 (6)	−0.0083 (5)	0.0017 (5)	0.0045 (5)
C12B	0.0392 (7)	0.0321 (7)	0.0328 (7)	−0.0080 (6)	−0.0024 (5)	−0.0022 (5)
C13B	0.0327 (7)	0.0401 (7)	0.0304 (6)	−0.0090 (6)	−0.0049 (5)	−0.0018 (6)
C14B	0.0225 (6)	0.0420 (7)	0.0306 (6)	−0.0049 (5)	0.0022 (5)	0.0015 (5)
C15B	0.0289 (6)	0.0392 (7)	0.0294 (6)	−0.0041 (5)	0.0050 (5)	−0.0061 (5)
C16B	0.0298 (6)	0.0426 (7)	0.0219 (6)	−0.0080 (6)	0.0009 (5)	−0.0017 (5)
C17B	0.0721 (12)	0.1183 (18)	0.0302 (8)	0.0216 (12)	−0.0096 (8)	0.0101 (10)
C18B	0.0637 (12)	0.0739 (13)	0.1000 (16)	0.0367 (11)	−0.0359 (11)	−0.0413 (12)

### Geometric parameters (Å, º)

**Table:**

O1A—C1A	1.4256 (14)	O1B—C1B	1.4239 (16)
O1A—H1O	0.89 (2)	O1B—H2O	0.88 (2)
O2A—C14A	1.3722 (14)	O2B—C14B	1.3705 (16)
O2A—C18A	1.4231 (16)	O2B—C18B	1.399 (2)
C1A—C2A	1.5059 (16)	C1B—C2B	1.506 (2)
C1A—C10A	1.5408 (16)	C1B—C10B	1.5333 (19)
C1A—H1AA	1.0000	C1B—H1BA	1.0000
C2A—C3A	1.3834 (17)	C2B—C3B	1.384 (2)
C2A—C7A	1.4087 (17)	C2B—C7B	1.406 (2)
C3A—C4A	1.387 (2)	C3B—C4B	1.386 (3)
C3A—H3AA	0.9500	C3B—H3BA	0.9500
C4A—C5A	1.374 (3)	C4B—C5B	1.372 (3)
C4A—H4AA	0.9500	C4B—H4BA	0.9500
C5A—C6A	1.383 (3)	C5B—C6B	1.388 (3)
C5A—H5AA	0.9500	C5B—H5BA	0.9500
C6A—C7A	1.401 (2)	C6B—C7B	1.396 (2)
C6A—H6AA	0.9500	C6B—H6BA	0.9500
C7A—C8A	1.474 (2)	C7B—C8B	1.472 (2)
C8A—C9A	1.332 (2)	C8B—C9B	1.329 (2)
C8A—C17A	1.5060 (19)	C8B—C17B	1.512 (2)
C9A—C10A	1.5095 (19)	C9B—C10B	1.5095 (19)
C9A—H9AA	0.9500	C9B—H9BA	0.9500
C10A—C11A	1.5201 (16)	C10B—C11B	1.5197 (19)
C10A—H10A	1.0000	C10B—H10B	1.0000
C11A—C16A	1.3891 (17)	C11B—C16B	1.3837 (19)
C11A—C12A	1.3956 (17)	C11B—C12B	1.4015 (18)
C12A—C13A	1.3817 (17)	C12B—C13B	1.370 (2)
C12A—H12A	0.9500	C12B—H12B	0.9500
C13A—C14A	1.3889 (17)	C13B—C14B	1.390 (2)
C13A—H13A	0.9500	C13B—H13B	0.9500
C14A—C15A	1.3877 (16)	C14B—C15B	1.3862 (18)
C15A—C16A	1.3900 (17)	C15B—C16B	1.3939 (19)
C15A—H15A	0.9500	C15B—H15B	0.9500
C16A—H16A	0.9500	C16B—H16B	0.9500
C17A—H17A	0.9800	C17B—H17D	0.9800
C17A—H17B	0.9800	C17B—H17E	0.9800
C17A—H17C	0.9800	C17B—H17F	0.9800
C18A—H18A	0.9800	C18B—H18D	0.9800
C18A—H18B	0.9800	C18B—H18E	0.9800
C18A—H18C	0.9800	C18B—H18F	0.9800
			
C1A—O1A—H1O	107.1 (14)	C1B—O1B—H2O	108.9 (15)
C14A—O2A—C18A	117.10 (9)	C14B—O2B—C18B	117.94 (12)
O1A—C1A—C2A	110.12 (9)	O1B—C1B—C2B	109.66 (11)
O1A—C1A—C10A	111.84 (9)	O1B—C1B—C10B	112.26 (11)
C2A—C1A—C10A	113.51 (10)	C2B—C1B—C10B	113.26 (11)
O1A—C1A—H1AA	107.0	O1B—C1B—H1BA	107.1
C2A—C1A—H1AA	107.0	C2B—C1B—H1BA	107.1
C10A—C1A—H1AA	107.0	C10B—C1B—H1BA	107.1
C3A—C2A—C7A	119.99 (11)	C3B—C2B—C7B	119.58 (14)
C3A—C2A—C1A	121.38 (11)	C3B—C2B—C1B	121.61 (13)
C7A—C2A—C1A	118.26 (11)	C7B—C2B—C1B	118.52 (12)
C2A—C3A—C4A	120.85 (13)	C2B—C3B—C4B	121.18 (17)
C2A—C3A—H3AA	119.6	C2B—C3B—H3BA	119.4
C4A—C3A—H3AA	119.6	C4B—C3B—H3BA	119.4
C5A—C4A—C3A	119.74 (14)	C5B—C4B—C3B	119.73 (17)
C5A—C4A—H4AA	120.1	C5B—C4B—H4BA	120.1
C3A—C4A—H4AA	120.1	C3B—C4B—H4BA	120.1
C4A—C5A—C6A	120.21 (13)	C4B—C5B—C6B	119.96 (17)
C4A—C5A—H5AA	119.9	C4B—C5B—H5BA	120.0
C6A—C5A—H5AA	119.9	C6B—C5B—H5BA	120.0
C5A—C6A—C7A	121.17 (14)	C5B—C6B—C7B	121.18 (18)
C5A—C6A—H6AA	119.4	C5B—C6B—H6BA	119.4
C7A—C6A—H6AA	119.4	C7B—C6B—H6BA	119.4
C6A—C7A—C2A	118.01 (13)	C6B—C7B—C2B	118.35 (15)
C6A—C7A—C8A	122.73 (13)	C6B—C7B—C8B	122.50 (14)
C2A—C7A—C8A	119.24 (11)	C2B—C7B—C8B	119.14 (13)
C9A—C8A—C7A	119.95 (11)	C9B—C8B—C7B	120.00 (12)
C9A—C8A—C17A	121.61 (15)	C9B—C8B—C17B	121.27 (16)
C7A—C8A—C17A	118.44 (14)	C7B—C8B—C17B	118.60 (15)
C8A—C9A—C10A	123.75 (12)	C8B—C9B—C10B	124.18 (13)
C8A—C9A—H9AA	118.1	C8B—C9B—H9BA	117.9
C10A—C9A—H9AA	118.1	C10B—C9B—H9BA	117.9
C9A—C10A—C11A	111.50 (10)	C9B—C10B—C11B	112.73 (12)
C9A—C10A—C1A	108.73 (10)	C9B—C10B—C1B	109.29 (11)
C11A—C10A—C1A	115.49 (10)	C11B—C10B—C1B	113.94 (10)
C9A—C10A—H10A	106.9	C9B—C10B—H10B	106.8
C11A—C10A—H10A	106.9	C11B—C10B—H10B	106.8
C1A—C10A—H10A	106.9	C1B—C10B—H10B	106.8
C16A—C11A—C12A	117.15 (11)	C16B—C11B—C12B	117.49 (12)
C16A—C11A—C10A	122.64 (11)	C16B—C11B—C10B	123.35 (11)
C12A—C11A—C10A	120.17 (11)	C12B—C11B—C10B	119.16 (12)
C13A—C12A—C11A	122.11 (11)	C13B—C12B—C11B	121.39 (13)
C13A—C12A—H12A	118.9	C13B—C12B—H12B	119.3
C11A—C12A—H12A	118.9	C11B—C12B—H12B	119.3
C12A—C13A—C14A	119.55 (11)	C12B—C13B—C14B	120.41 (12)
C12A—C13A—H13A	120.2	C12B—C13B—H13B	119.8
C14A—C13A—H13A	120.2	C14B—C13B—H13B	119.8
O2A—C14A—C15A	124.33 (11)	O2B—C14B—C15B	125.22 (12)
O2A—C14A—C13A	115.97 (10)	O2B—C14B—C13B	115.31 (11)
C15A—C14A—C13A	119.70 (11)	C15B—C14B—C13B	119.47 (12)
C14A—C15A—C16A	119.71 (11)	C14B—C15B—C16B	119.41 (12)
C14A—C15A—H15A	120.1	C14B—C15B—H15B	120.3
C16A—C15A—H15A	120.1	C16B—C15B—H15B	120.3
C11A—C16A—C15A	121.74 (11)	C11B—C16B—C15B	121.81 (12)
C11A—C16A—H16A	119.1	C11B—C16B—H16B	119.1
C15A—C16A—H16A	119.1	C15B—C16B—H16B	119.1
C8A—C17A—H17A	109.5	C8B—C17B—H17D	109.5
C8A—C17A—H17B	109.5	C8B—C17B—H17E	109.5
H17A—C17A—H17B	109.5	H17D—C17B—H17E	109.5
C8A—C17A—H17C	109.5	C8B—C17B—H17F	109.5
H17A—C17A—H17C	109.5	H17D—C17B—H17F	109.5
H17B—C17A—H17C	109.5	H17E—C17B—H17F	109.5
O2A—C18A—H18A	109.5	O2B—C18B—H18D	109.5
O2A—C18A—H18B	109.5	O2B—C18B—H18E	109.5
H18A—C18A—H18B	109.5	H18D—C18B—H18E	109.5
O2A—C18A—H18C	109.5	O2B—C18B—H18F	109.5
H18A—C18A—H18C	109.5	H18D—C18B—H18F	109.5
H18B—C18A—H18C	109.5	H18E—C18B—H18F	109.5
			
O1A—C1A—C2A—C3A	−25.34 (14)	O1B—C1B—C2B—C3B	−22.59 (17)
C10A—C1A—C2A—C3A	−151.59 (11)	C10B—C1B—C2B—C3B	−148.85 (12)
O1A—C1A—C2A—C7A	161.64 (10)	O1B—C1B—C2B—C7B	163.61 (11)
C10A—C1A—C2A—C7A	35.38 (14)	C10B—C1B—C2B—C7B	37.35 (16)
C7A—C2A—C3A—C4A	−0.87 (18)	C7B—C2B—C3B—C4B	1.4 (2)
C1A—C2A—C3A—C4A	−173.78 (11)	C1B—C2B—C3B—C4B	−172.34 (14)
C2A—C3A—C4A—C5A	−0.5 (2)	C2B—C3B—C4B—C5B	0.0 (2)
C3A—C4A—C5A—C6A	1.2 (2)	C3B—C4B—C5B—C6B	−1.1 (3)
C4A—C5A—C6A—C7A	−0.6 (2)	C4B—C5B—C6B—C7B	0.8 (3)
C5A—C6A—C7A—C2A	−0.68 (19)	C5B—C6B—C7B—C2B	0.5 (2)
C5A—C6A—C7A—C8A	178.05 (12)	C5B—C6B—C7B—C8B	−178.57 (15)
C3A—C2A—C7A—C6A	1.42 (17)	C3B—C2B—C7B—C6B	−1.6 (2)
C1A—C2A—C7A—C6A	174.54 (11)	C1B—C2B—C7B—C6B	172.31 (12)
C3A—C2A—C7A—C8A	−177.36 (11)	C3B—C2B—C7B—C8B	177.51 (13)
C1A—C2A—C7A—C8A	−4.23 (16)	C1B—C2B—C7B—C8B	−8.56 (18)
C6A—C7A—C8A—C9A	166.49 (12)	C6B—C7B—C8B—C9B	167.58 (15)
C2A—C7A—C8A—C9A	−14.79 (18)	C2B—C7B—C8B—C9B	−11.5 (2)
C6A—C7A—C8A—C17A	−14.08 (19)	C6B—C7B—C8B—C17B	−8.4 (2)
C2A—C7A—C8A—C17A	164.64 (13)	C2B—C7B—C8B—C17B	172.50 (15)
C7A—C8A—C9A—C10A	0.12 (19)	C7B—C8B—C9B—C10B	0.5 (2)
C17A—C8A—C9A—C10A	−179.30 (13)	C17B—C8B—C9B—C10B	176.41 (16)
C8A—C9A—C10A—C11A	−98.56 (14)	C8B—C9B—C10B—C11B	−100.29 (17)
C8A—C9A—C10A—C1A	29.90 (17)	C8B—C9B—C10B—C1B	27.5 (2)
O1A—C1A—C10A—C9A	−171.04 (10)	O1B—C1B—C10B—C9B	−169.13 (12)
C2A—C1A—C10A—C9A	−45.70 (13)	C2B—C1B—C10B—C9B	−44.26 (15)
O1A—C1A—C10A—C11A	−44.85 (14)	O1B—C1B—C10B—C11B	−42.02 (16)
C2A—C1A—C10A—C11A	80.49 (13)	C2B—C1B—C10B—C11B	82.85 (14)
C9A—C10A—C11A—C16A	53.71 (15)	C9B—C10B—C11B—C16B	31.98 (17)
C1A—C10A—C11A—C16A	−71.05 (15)	C1B—C10B—C11B—C16B	−93.32 (15)
C9A—C10A—C11A—C12A	−123.89 (12)	C9B—C10B—C11B—C12B	−148.38 (13)
C1A—C10A—C11A—C12A	111.34 (12)	C1B—C10B—C11B—C12B	86.32 (15)
C16A—C11A—C12A—C13A	−0.81 (17)	C16B—C11B—C12B—C13B	−0.52 (19)
C10A—C11A—C12A—C13A	176.92 (11)	C10B—C11B—C12B—C13B	179.82 (12)
C11A—C12A—C13A—C14A	−0.07 (18)	C11B—C12B—C13B—C14B	−0.4 (2)
C18A—O2A—C14A—C15A	−1.38 (16)	C18B—O2B—C14B—C15B	4.6 (2)
C18A—O2A—C14A—C13A	178.12 (11)	C18B—O2B—C14B—C13B	−175.02 (16)
C12A—C13A—C14A—O2A	−178.15 (10)	C12B—C13B—C14B—O2B	−179.59 (12)
C12A—C13A—C14A—C15A	1.37 (17)	C12B—C13B—C14B—C15B	0.76 (19)
O2A—C14A—C15A—C16A	177.74 (11)	O2B—C14B—C15B—C16B	−179.77 (11)
C13A—C14A—C15A—C16A	−1.75 (17)	C13B—C14B—C15B—C16B	−0.15 (18)
C12A—C11A—C16A—C15A	0.42 (17)	C12B—C11B—C16B—C15B	1.14 (18)
C10A—C11A—C16A—C15A	−177.25 (11)	C10B—C11B—C16B—C15B	−179.21 (12)
C14A—C15A—C16A—C11A	0.85 (18)	C14B—C15B—C16B—C11B	−0.82 (19)

### Hydrogen-bond geometry (Å, º)

**Table:**

*D*—H···*A*	*D*—H	H···*A*	*D*···*A*	*D*—H···*A*
O1*A*—H1*O*···O1*B*	0.89 (2)	2.23 (2)	3.0346 (15)	150.6 (19)
O1*B*—H2*O*···O1*A*^i^	0.88 (2)	2.04 (2)	2.8973 (15)	163 (2)

Symmetry code: (i) −*x*+1/2, *y*−1/2, *z*.
